# The importance of LDL-C lowering in atherosclerotic cardiovascular disease prevention: Lower for longer is better

**DOI:** 10.1016/j.ajpc.2024.100649

**Published:** 2024-03-18

**Authors:** Omar Mhaimeed, Zain A Burney, Stacey L Schott, Payal Kohli, Francoise A Marvel, Seth S Martin

**Affiliations:** a Department of Medicine, Johns Hopkins Hospital, Baltimore, MD, United States; b Department of Medicine, Cleveland Clinic, Cleveland, OH, United States; c Ciccarone Center for the Prevention of Cardiovascular Disease, Division of Cardiology, Johns Hopkins University School of Medicine, Baltimore, MD, United States; d Department of Cardiology, University of Colorado Anschutz, Aurora, CO, United States; e Department of Cardiology, Veterans Affairs Hospital, Aurora, CO, United States; f Cherry Creek Heart, Aurora, CO, United States; g Tegna Broadcasting, MD, United States

**Keywords:** LDL cholesterol, Lipid lowering, Statins, Non-statin, Primary prevention, Secondary prevention, Atherosclerotic cardiovascular disease

## Abstract

Cumulative exposure to low-density lipoprotein cholesterol (LDL-C) is a key driver of atherosclerotic cardiovascular disease (ASCVD) risk. An armamentarium of therapies to achieve robust and sustained reduction in LDL-C can reduce ASCVD risk. The gold standard for LDL-C assessment is ultracentrifugation but in routine clinical practice LDL-C is usually calculated and the most accurate calculation is the Martin/Hopkins equation. For primary prevention, consideration of estimated ASCVD risk frames decision making regarding use of statins and other therapies, and tools such as risk enhancing factors and coronary artery calcium enable tailoring of risk assessment and decision making. In patients with diabetes, lipid lowering therapy is recommended in most patients to reduce ASCVD risk with an opportunity to tailor therapy based on other risk factors. Patients with primary hypercholesterolemia and familial hypercholesterolemia (FH) with baseline LDL-C greater than or equal to 190 mg/dL are at elevated risk, and LDL-C lowering with high-intensity statin therapy is often combined with non-statin therapies to prevent ASCVD. Secondary prevention of ASCVD, including in patients with prior myocardial infarction or stroke, requires intensive lipid lowering therapy and lifestyle modification approaches. There is no established LDL-C level below which benefit ceases or safety concerns arise. When further LDL-C lowering is required beyond lifestyle modifications and statin therapy, additional medications include oral ezetimibe and bempedoic acid, or injectables such as PCSK9 monoclonal antibodies or siRNA therapy. A novel agent that acts independently of hepatic LDL receptors is evinacumab, which is approved for patients with homozygous FH. Other emerging agents are targeted at Lp(a) and CETP. In light of the expanding lipid treatment landscape, this manuscript reviews the importance of early, intensive, and sustained LDL-C-lowering for primary and secondary prevention of ASCVD.

## Graphical abstract

**Figure:**
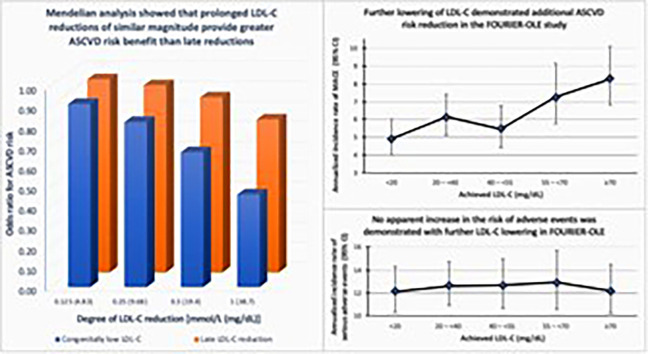

## 1 Introduction

Mounting evidence shows that when lowering low-density lipoprotein cholesterol (LDL-C) to prevent atherosclerotic cardiovascular disease (ASCVD), lower for longer is better [1]. The risk of ASCVD is closely correlated with cumulative exposure to LDL-C, that is, the magnitude of elevation in LDL-C multiplied by the years of association, coined as the “cholesterol years” of exposure [2], [3], [4], [5]. In light of the evolving therapeutic landscape for dyslipidemia, this manuscript reviews the importance of early LDL-C-lowering for primary and secondary ASCVD prevention, with an overview of established and newer LDL-C lowering drugs.

### 1.1 Importance of lowering LDL-C for prevention of ASCVD

LDL-C has been the most commonly used clinical lipid measure in ASCVD prevention. The clinical definition of LDL-C is non-HDL-C minus VLDL-C, thus clinical LDL-C includes biologic LDL-C + IDL-C + Lp(a)-C. The Friedewald equation, developed in 1972, estimates LDL-C as: total cholesterol (TC) minus high-density lipoprotein cholesterol (HDL-C) minus triglycerides (TG)/5 in mg/dL, with the latter function estimating very low-density lipoprotein-cholesterol [6]. At low LDL-C or high TG levels, this equation is vulnerable to inaccuracies, in particular underestimation of LDL-C [7,8]. The gold standard of LDL-C measurement is ultracentrifugation, but in routine clinical practice, it is usually calculated from the standard lipid panel, with the most accurate calculation being the Martin/Hopkins equation [9], [10], [11].

The Framingham Heart Study identified cholesterol as a risk factor associated with coronary artery disease (CAD) in 1961 and since then, growing clinical, epidemiologic, and randomized trial evidence has firmly established LDL-C as a causal and highly modifiable risk factor in the development and pathogenesis of ASCVD [12,13]. ASCVD may present (clinically or subclinically) as CAD, peripheral arterial disease, and cerebrovascular disease [14]. According to the American Heart Association (AHA) Annual Heart Disease and Stroke Statistical Update, between 2017 and 2020 the prevalence of CAD among adults was 20.5 million and the prevalence of stroke was 9.4 million [14].

Between 2017 to 2020, the mean LDL-C for adults above 20 years of age living in the United States was 110 mg/dL. The age-adjusted prevalence of high LDL-C (defined as ≥130 mg/dL) was 25.5%. Among adults in the same time frame, LDL-C ≥ 130 mg/dL occurred in 25.6% of males and 25.4% of females. In 2020, age-adjusted death rates due to CAD per 100,000 were 128.5 for non-Hispanic white males, 153.6 for non-Hispanic Black males, and 102.2 for Hispanic males. For non-Hispanic white females, it was 63.8, 85.9 for non-Hispanic Black females, and 54.2 for Hispanic females [14]. Furthermore, in a National Inpatient Sample (NIS) analysis of sex differences in patients hospitalized for acute myocardial infarction spanning 2004 to 2015, compared with males, females had a higher odds of all-cause mortality (aOR, 1.03; 95% CI, 1.02 – 1.04; *p* < 0.001), with similar observations recorded for major adverse cardiovascular events [15].

Among the Global Burden of Disease (GBD) data, the global years of life lost (YLL) attributable to high LDL-C totaled 4.51 (95% UI, 2.65 – 6.24) in 2020, and the population attributable factor (PAF) was 7.96% (95% UI, 4.68% – 11.02%). LDL-C was also the third highest contributor to cardiovascular disease disability-adjusted life years (CVD DALY), after systolic blood pressure and dietary risks [16]. The role of LDL-C lowering to reduce ASCVD risk is one of the most investigated and highly established relationships in modern medicine [17,2]. Every 1 mmol/L (∼39 mg/dL) reduction in LDL-C correlates with a 20–25% reduction in risk of cardiovascular events [13,18,19].

### 1.2 Mechanisms by which LDL-C contributes to ASCVD

The precursor to LDL is VLDL, which carries triglycerides and cholesterol synthesized in the liver through the circulation. As triglycerides within VLDL are metabolized, the lipoprotein becomes denser, forming LDL, which is cleared from the circulation by hepatic reuptake [20]. Atherogenesis occurs as cholesterol from LDL pathologically builds up in arterial walls, where it is subsequently engulfed by macrophages and oxidized. Clinically significant plaques may either limit blood flow, resulting in angina and demand ischemia, or may rupture and thrombose, resulting in ischemia (myocardial infarction, or MI) [21].

**Fig. 1:**
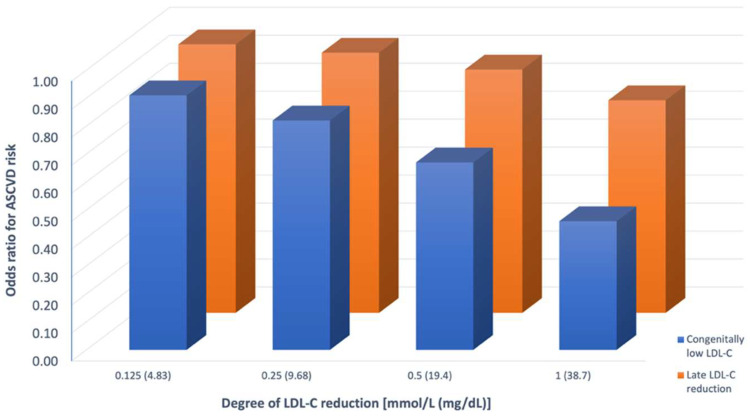
**Lower LDL-C for longer periods of time is better**[15]. Lower LDL-C sustained for longer periods of time is associated with lower odds of ASCVD. The X axis represents the degree of absolute LDL-C reduction in two subgroups: those with congenitally low LDL-C, and those who were started on medical therapy later in life. Those with congenitally low LDL-C levels have lower odds of ASCVD with a similar degree of LDL-C reduction as compared to those started on pharmacologic therapy later in life.

The observation that ASCVD risk is a function of the cumulative exposure to LDL-C is supported by studies showing that long-term exposure to congenitally lower LDL-C is associated with a greater reduction in ASCVD risk per unit reduction in LDL-C compared with shorter-term lowering via pharmacotherapy, and with additional scientific evidence demonstrating increasing benefit observed over time in statin treated patients **(**Fig. 1**)** [1,22,23].

### 1.3 LDL-C management for primary prevention

Primary prevention guidelines aim to match estimated risk with the recommended intensity of LDL-C lowering. In the AHA/ACC guidelines, the Pooled Cohort Equation (PCE) is used to estimate 10-year ASCVD risk to guide decision making about preventive interventions. The PCE is intended for adults 40 to 75 years of age with LDL-C above 70 mg/dL but less than 190 mg/dL. The 10-year ASCVD risk categories are: low (<5%), borderline (5 to <7.5%), intermediate (≥7.5% to <20%), or high (≥20%) [24,25]. Primary prevention in those above 75 years requires shared decision-making with consideration of competing risk from comorbidities and overall life expectancy [24]. The ESC guidelines also utilize a 10-year risk-based approach to prevention that incorporates the SCORE2 and SCORE2-OP (older persons) risk algorithms to create categories of risk according to age [26].

All risk estimation tools have limitations, however. Notable risk factors used in the PCE include age, sex, race, total cholesterol, HDL cholesterol, systolic blood pressure, treatment for hypertension, and diabetes, but it may over or underestimate risk for certain subgroups such as those with obesity, Black adults, and South Asian adults [24,27,28,29]. Factors such as family history, social determinants of health, and physical activity are not captured. Review of additional risk-enhancing factors should be done to refine PCE risk assessment and help tailor decision making [30]. The list of risk-enhancing factors includes family history, kidney disease, metabolic syndrome, preeclampsia, early menopause, inflammatory conditions, ethnicity, elevated Lp(a) or apoB, and coronary artery calcium (CAC) scoring, among others. Furthermore, younger individuals <age 40 years old do not qualify for use of the PCE. Newer adjunct risk stratification strategies such as CAC scoring and PRS may help tailor statin prescriptions in this group [31,32].

Counseling on lifestyle modifications is imperative for all patients. It may be the primary strategy for select patients, particularly those at lower 10-year or lifetime risk of ASCVD. Lifestyle modifications include a healthy diet, regular moderate intensity exercise, weight management, and smoking cessation.

Among those with intermediate risk (≥7.5% to <20%), the initiation of a moderate or high-intensity statin to reduce LDL-C by 30% or more, in addition to the adoption of a healthy lifestyle is recommended. The choice of a moderate versus high-intensity statin depends on the overall risk profile of the patient [24]. The AHA/ACC guidelines recognize an opportunity to tailor decision making in this sub-group using atherosclerosis imaging and risk-enhancing factors. It may be reasonable for these patients to undergo CAC scoring to assess their atherosclerotic burden [24]. Patients with a non-zero CAC score, particularly those with a score >100, are recommended for statin therapy. Those with a CAC score of 0 can consider deferring statin therapy after shared decision making, unless they are an individual who smokers, has diabetes, or has a history of familial hypercholesterolemia (FH) [24]. Aside from CAC, the presence of risk-enhancing factors suggests starting or intensifying primary preventive therapies in those at intermediate or borderline risk [24,30]. In those at highest risk (>20%), the use of a high-intensity statin to reduce the LDL-C by 50% or more is recommended in addition to a healthy lifestyle [24].

The AHA/ACC and ESC guidelines concur that maintaining LDL-C < 100 mg/dL is generally desired in the primary prevention setting. However, levels well below 100 mg/dL may be desirable depending on the primary prevention context, including the presence of multiple risk modifiers. Per ESC guidelines, depending on the 10-year ASCVD risk ascertained by the SCORE2 calculator, comorbidities, frailty, and patient preferences, treatment can be intensified to target LDL-C levels below 70 mg/dL in those at high risk, or below 55 mg/dL if at very high risk [26].

## 2 High-risk primary prevention

### 2.1 Diabetes

For patients with diabetes between ages 40 to 75 years and with an LDL-C level ≥70 mg/dL, guidelines recommend initiation of a moderate-intensity statin [24]. Furthermore, for those with diabetes and multiple ASCVD risk factors, a high-intensity statin to reduce LDL-C by 50% or more is recommended [24]. In those with a 10-year risk estimated >20%, the addition of ezetimibe can be considered for lower LDL-C thresholds in primary prevention [24]. In adults older than 75 years who are already on a statin, it is reasonable to continue therapy whereas new initiation of therapy should be based on clinician-patient discussion [24]. Patients with diabetes aged 20–39 years may be considered for statin therapy based on duration of diabetes, renal markers, and other risk factors [24].

The ESC guidelines call for patients with diabetes and without ASCVD or other severe target organ damage but at high risk to target LDL-C levels below 100 mg/dL. Treatment can be further intensified to reduce LDL-C levels below 70 mg/dL, depending on their estimated 10-year risk, lifetime ASCVD risk, comorbidities, glycemic control, frailty, and patient preferences. Those at moderate risk are not recommended for additional prevention goals [26].

### 2.2 Severe primary hypercholesterolemia

Severe primary hypercholesterolemia is defined as an LDL-C of 190 mg/dL or above. These patients have a high risk of ASCVD and do not require 10-year risk estimation to initiate pharmacologic intervention. Since high-intensity statin therapy provides greater ASCVD risk reduction than moderate-intensity statin, maximally tolerated statin therapy should be prescribed to patients with an LDL-C of 190 mg/dL or above. If the on-treatment LDL-C levels remain at 100 mg/dL or above, adding ezetimibe is reasonable [25]. If maximally tolerated statin therapy and ezetimibe fail to reduce the LDL-C below 100 mg/dL, a PCSK9 inhibitor may be considered [24].

LDL-C levels ≥190 mg/dL or ≥160 mg/dL coupled with a family history of premature CAD should prompt further evaluation for FH [33]. FH is the most common monogenic lipid disorder affecting an estimated 1:250 people worldwide, and is marked by accelerated atherosclerosis due to the cumulative lifetime exposure to high levels of LDL-C [34]. Due to their high starting LDL-C level, patients with FH typically require combination lipid lowering therapy with statin and non-statin therapy [24].

### 2.3 Cardiovascular kidney-metabolic syndrome

Cardiovascular-kidney-metabolic syndrome has been conceptualized as a result of the growing appreciation of the interrelationship between metabolic risk factors, chronic kidney disease, and cardiovascular disease. Patients with cardiovascular disease and concomitant metabolic risk factors with or without chronic kidney disease should be approached with unique considerations. The Scientific Statement from the American Heart Association stratifies individuals with this syndrome into five stages, from Stage 0 to Stage 4, with each stage calling for its own set of recommendations [35,36]. Stage 0 includes those without CKM risk factors, stage 1 encompasses those with excess or dysfunctional adiposity, and stage 2 includes patients with metabolic risk factors or chronic kidney disease. Once patients develop subclinical cardiovascular disease overlapping with CKM risk factors, they are in stage 3, and they are in stage 4 once they have clinical cardiovascular disease overlapping with CKM risk factors [35,36]. In addition to lipid-lowering therapies, the CKM advisory recommends selecting cardioprotective antihyperglycemic agents among those with diabetes, and SGLT2 inhibitors (SGLT2i) for those with CKD, existing heart failure, or those at high risk of heart failure [35,36]. GLP1 receptor agonists (GLP1 RA) are indicated for those with uncontrolled hyperglycemia, or severe obesity/insulin resistance [35,36]. Combined use of both SGLT2i and GLP1 RA is considered for those with multiple CKM risk factors in the setting of cardiovascular disease or high predicted risk of cardiovascular disease [35,36].

As a result of the CKM construct, the PREVENT risk calculator has been conceived and is intended for primary prevention patients between the ages of 30 and 79 years [37,38]. The equations were derived and validated in a large sample of over 6 million individuals [37,38]. The calculator includes kidney and metabolic inputs, social deprivation index input and has heart failure risk and 30-year risk added as an output [37,38]. It has been tested across varied racial and ethnic groups and had similar accuracy [37,38]. The inclusion of individuals as young as 30 years old may help in addressing the age limitation of the PCE noted above. However, further studies are needed to help understand how output from the PREVENT risk calculator can best be applied in clinical management.

## 3 LDL-C management for secondary prevention

Pharmacotherapy for LDL-C lowering in secondary prevention based on AHA/ACC guidelines is guided by phenotyping patients as “very high risk”, characterized by recurrent ASCVD events or by one ASCVD event with multiple high-risk conditions (e.g., hypertension, kidney disease, diabetes). For those at very high risk, regardless of age, high-intensity statin therapy or the maximum tolerated intensity is recommended. If on-treatment LDL-C remains ≥70 mg/dL, non-statin therapy with ezetimibe and/or a PCSK9 inhibitor warrants consideration. In those who are not very high risk and aged ≤75 years with clinical ASCVD, high-intensity statin therapy is recommended as the first-line lipid lowering therapy to reduce LDL-C levels by ≥50%. If on-treatment LDL-C is ≥70 mg/dL, ezetimibe may be considered. In those not very high risk and >75 years of age with ASCVD, moderate- or high-intensity statin therapy should be continued or initiated.

Once a patient has been initiated on LDL-C lowering therapy, assessment of adherence to medications and lifestyle modifications is crucial. The percentage lowering of LDL-C should be assessed with repeat lipid measurements within 4–12 weeks after medication initiation or dose adjustment and repeated every 3–12 months thereafter [24,25]. The European guidelines and the ACC Non-Statin Consensus Pathway recommend an LDL-C level <55 mg/dL in patients with established ASCVD, or in adults with clinical ASCVD at very high risk on statin therapy, respectively, as there appears to be no LDL-C level below which reduction of cardiovascular events ceases [26,39].

## 4 Nonpharmacologic management of LDL-C

Lifestyle interventions to reduce LDL-C and improve cardiovascular health are a foundation of therapy. Regarding diet, consumption of unsaturated is preferred versus saturated and *trans* fats for managing LDL-C [40,41]. The AHA/ACC guidelines advise consumption of a dietary pattern that emphasizes intake of vegetables, fruits, whole grains, legumes, healthy protein sources (low-fat dairy products, low-fat poultry (without the skin), fish/seafood, and nuts), and nontropical vegetable oils; and limits intake of sweets, sugar-sweetened beverages, and red meats. This dietary pattern should be tailored to the individual based on caloric requirements and preferences. In terms of physical activity and exercise, the adoption of 40 min sessions of moderate-to vigorous intensity exercise 3–4 times per week is also recommended [24].

## 5 Pharmacologic management of LDL-C

In this section, we discuss the widely used pharmacotherapies to lower LDL-C in the order in which they were FDA approved and introduced into clinical practice. We begin with discussion of statins, followed by ezetimibe, PCSK9 monoclonal antibodies, bempedoic acid, and small interfering RNA against PCSK9, with a subsection discussing the safety of very low LDL-C. We then also discuss two therapies which are approved for use in homozygous familial hypercholesterolemia (HoFH), namely lomitapide and evinacumab.

### 5.1 Statins

Among medical therapies to reduce serum LDL-C, inhibitors of an enzyme in the cholesterol biosynthesis pathway called hydroxymethylglutaryl-coenzyme A (HMG-CoA) reductase known as statins, are first line for both primary and secondary prevention based on a wealth of evidence (Table 1). In 1994, simvastatin was evaluated for the secondary prevention of CAD. Simvastatin outperformed placebo by a 3.3% absolute risk reduction (ARR) (RRR 30%; 95% CI, 15% to 42%) in all-cause mortality in the 4S trial, and reduced major coronary events, coronary intervention, and cardiovascular mortality [42]. It reduced LDL-C by 35% at one year and 38% at three years. The following year, the WOSCOPS trial of pravastatin versus placebo demonstrated a primary prevention benefit in men with hyperlipidemia, defined as a fasting LDL-C level of 155 mg/dL or more despite appropriate dietary strategies [43]. The trial demonstrated a 2.4% ARR (RRR 31%; 95% CI, 17% to 43%) in the composite of nonfatal MI and CAD death, as well as a 32% RRR in cardiovascular mortality, and LDL-C was reduced by 26% at 5 years. Simvastatin also outperformed placebo in the large HPS trial of 20,536 high-risk patients with or without ASCVD. At a median 5 year follow-up, it demonstrated a 29% LDL-C reduction and a 1.8% ARR in all-cause mortality (RRR 13%; 95% CI, 6% to 19%) [44].

**Table 1:** Randomized controlled trials of statin drugs in various populations.

TRIAL	POPULATION	SAMPLE SIZE	INTERVENTION	CONTROL	PRIMARY OUTCOME COMPOSITION	LDL-C REDUCTION	EFFECT ON PRIMARY OUTCOME
4S	Stable CAD	4,444	Simvastatin	Placebo	ACM	35%	RR 0.70 (0.58–0.85)
WOSCOPS	Men with LDL ≥ 155	6,595	Pravastatin 40 mg	Placebo	MI, CVM	26%	RR 0.69 (0.57–0.83)
HPS	ASCVD, HR-Primary	20,536	Simvastatin 40 mg	Placebo	ACM	29%	RR 0.87 (0.81–0.94)
PROVE IT-TIMI 22	Post-ACS	4,162	Atorvastatin 80 mg	Pravastatin 40 mg	MI, St, UA-H, ReV, ACM	32%	HR 0.84 (0.74–0.95)
CARDS	T2DM	2,383	Atorvastatin 10 mg	Placebo	ACS, St, ReV	40%	HR 0.63 (0.48–0.83)
TNT	Stable CAD	10,001	Atorvastatin 80 mg	Atorvastatin 10 mg	MI, St, CA-R, CVM	24%	HR 0.78 (0.69–0.89)
JUPITER	Elevated hs-CRP	17,802	Rosuvastatin 20 mg	Placebo	MI, St, UA-H, ReV, CVM	50%	HR 0.56 (0.46–0.69)
REPRIEVE	HIV infection	7,769	Pitavastatin 4 mg	Placebo	MI, St, TIA, UA-H, ReV, CVM, UCM	31%	HR 0.65 (0.48–0.90)

4S: Scandinavian Simvastatin Survival Study; WOSCOPS: West of Scotland Prevention Study; HPS: Heart Protection Study; PROVE IT-TIMI 22: Pravastatin or Atorvastatin and Infection Therapy-Thrombolysis in Myocardial Infarction 22; CARDS: Collaborative Atorvastatin Diabetes Study; TNT: Treating to New Targets; JUPITER: Justification for the Use of Statins in Prevention - Intervention Trial Evaluating Rosuvastatin; REPRIEVE: Randomized Trial to Prevent Vascular Events in HIV. CAD: coronary artery disease; LDL: low-density lipoprotein; ASCVD: atherosclerotic cardiovascular disease; ACS: acute coronary syndrome; T2DM: type 2 diabetes mellitus; hs-CRP: high-sensitivity C reactive protein; HIV: human immunodeficiency virus; MI: myocardial infarction; St: stroke; TIA: transient ischemic attack; UA-H: unstable angina requiring hospitalization; ReV: arterial revascularization procedure; CA-R: resuscitated cardiac arrest; CVM: cardiovascular mortality; ACM: all-cause mortality; UCM: unknown cause mortality.

Over the next decade, trials were conducted using high-intensity statin therapy which resulted in an LDL-C reduction greater than 50%. The 2004 PROVE IT-TIMI 22 trial of atorvastatin 80 mg versus pravastatin 40 mg for secondary prevention demonstrated a 3.9% ARR (RRR 16%; 95% CI, 5% to 26%) for atorvastatin in the composite endpoint of all-cause mortality, MI, unstable angina (UA) rehospitalization, 30-day revascularization, and stroke [45]. At the conclusion of the study, the mean LDL-C in the atorvastatin group was 62 mg/dL versus 95 mg/dL in the pravastatin group. The CARDS trial randomized 2,383 patients with diabetes to receive either atorvastatin 10 mg or placebo. Over a median follow-up of 3.9 years, the atorvastatin group demonstrated a 4.1% ARR (HR 0.63; 95% CI, 0.48 to 0.83) in the primary composite outcome of ACS, coronary revascularization, and stroke [46].

The benefit of high intensity statin therapy compared to lower intensity statin therapy was redemonstrated in the TNT trial of atorvastatin. The trial randomized 10,001 patients with CAD to either atorvastatin 80 mg or atorvastatin 10 mg and demonstrated an ARR of 2.2% (HR 0.78; 95% CI, 0.69 to 0.89) in the composite outcome of CAD mortality, nonfatal MI, resuscitation after cardiac arrest, and stroke. This effect was primarily driven by reductions in non-fatal MI and stroke. The mean end-of-study LDL-C was 77 mg/dL in the high-intensity group (80 mg) versus 101 mg/dL in the moderate-intensity group (10 mg).

Trial data have shown a role for statin therapy in primary prevention even for certain groups of patients with well controlled LDL-C. The JUPITER trial randomized 17,802 patients with elevated hs-CRP (defined as > 2 mg/L) to rosuvastatin or placebo, and the median LDL-C at the start of the trial was 108 mg/dL [47]. Rosuvastatin significantly reduced both LDL-C (by 50%) and hs-CRP (by 37%), and demonstrated an absolute reduction in the primary major adverse cardiac event (MACE) endpoint of 0.59 events per 100 person-years (HR 0.56; 95% CI, 0.46 to 0.69), with reductions seen in all its components and in all-cause mortality. Most recently, the REPRIEVE trial of pitavastatin in 7,769 patients with HIV infection, who are higher risk for ASCVD also successfully demonstrated a MACE benefit of 2.51 events per 1000 person-years over placebo (HR 0.65; 95% CI 0.48–0.90) [48].

### 5.2 Ezetimibe

Ezetimibe directly inhibits cholesterol absorption at the intestinal brush border by inhibition of NPC1L1. The benefit of adjunctive ezetimibe was demonstrated in the IMPROVE-IT trial, which randomized 18,144 post-ACS patients to simvastatin in conjunction with either ezetimibe or placebo, and followed outcomes over a median of 6 years [49]. The combination-therapy group had a 2.0% ARR (HR 0.94; 95% CI, 0.89 to 0.99) in the primary composite outcome, which appeared to be driven primarily by a reduction in nonfatal MI and 30-day urgent revascularization. LDL-C was reduced by 24% in the ezetimibe group compared with placebo.

Ezetimibe is typically used in combination with statins, PCSK9 inhibitors, and other lipid lowering therapies in practice. There is a fixed dose combination pill of ezetimibe and bempedoic acid that recently became available. This is of particular interest since the cardiovascular outcomes trial (CLEAR-OUTCOMES) of bempedoic acid demonstrated reduction in MACE events in a statin-intolerant patient population [50].

### 5.3 PCSK9 monoclonal antibodies

PCSK9 monoclonal antibodies promote enhanced recycling of LDL receptors to the hepatocyte cell surface [51]. They are an FDA-approved adjunctive lipid lowering therapy used in both primary and secondary prevention of ASCVD, and supported by multiple randomized controlled trials (Table 2). The FOURIER study randomized 27,564 patients with stable CAD, but with multiple high-risk features who were maintained on a statin, to evolocumab every 2 or 4 weeks versus placebo [52]. Over a median of 2.2 years, evolocumab therapy resulted in an average of 59% reduction in LDL-C and a 1.5% ARR (HR 0.85; 95% CI, 0.79 to 0.92) in the primary composite outcome, driven primarily by a 1.2% ARR (HR 0.73) in MI [52]. The open-label FOURIER-OLE extension study followed these patients for a median of 5.0 additional years (longest follow up was >8 years) and demonstrated a 0.22% ARR (HR 0.77; 95% CI, 0.60 to 0.99) in cardiovascular mortality [53]. Notably, there was no tachyphylaxis or attenuation of efficacy of LDL-C reduction over time with evolocumab and no new or concerning safety signals with prolonged LDL-C lowering [53]. Similar findings were demonstrated in the ODYSSEY OUTCOMES trial of alirocumab, which randomized 18,924 patients who had a history of ACS within the past year to either biweekly alirocumab injections or placebo [54]. Over a median follow-up of 2.8 years, alirocumab demonstrated a 1.6% ARR (HR 0.85; 95% CI, 0.78 to 0.93) in the primary composite endpoint of CV death, MI, stroke, and hospitalization for UA, with all-cause mortality [54]. Most recently, the novel PCSK9i tafolecimab produced favorable LDL-C reductions in the Chinese CREDIT-2 trial, which randomized 149 patients with heterozygous familial hypercholesterolemia (HeFH) and followed them over 12 weeks [55].

**Table 2:** Randomized controlled trials of nonstatin lipid-lowering therapies in various populations.

TRIAL	POPULATION	SAMPLE SIZE	INTERVENTION	CONTROL	BACKGROUND THERAPY	PRIMARY OUTCOME COMPOSITION	LDL-C REDUCTION	EFFECT ON PRIMARY OUTCOME
IMPROVE-IT	Post-ACS	18,144	Simvastatin 40 mg-ezetimibe 10 mg	Simvastatin 40 mg	–	MI,St, UA-H, ReV, CVM	24%	HR 0.94 (0.89–0.99)
FOURIER	ASCVD	27,564	Evolocumab 140–420 mg	Placebo	Statin, 100%; ezetimibe, 5%	MI,St, UA-H, ReV, CVM	59%	HR 0.85 (0.79–0.92)
ODYSSEY OUTCOMES	Post-ACS	18,924	Alirocumab 75 mg	Placebo	High-intensity statin, 89%; other statin 8.5%; no statin 2.5%; ezetimibe 3%	MI, St, UA-H, CVM	55%	HR 0.85 (0.78–0.93)
CLEAR OUTCOMES	ASCVD, HR-Primary	13,970	Bempedoic acid 180 mg	Placebo	Statin, 23%; ezetimibe 12%	MI, St, ReV, CVM	21%	HR 0.87 (0.79–0.96)
ACCORD LIPID	T2DM	5,518	Fenofibrate 160 mg	Placebo	Simvastatin 20–40 mg, 100%	MI, St, CVM	NS	HR 0.92 (0.79–1.08)
HPS2-THRIVE	ASCVD	25,673	Niacin 1 g-laropiprant 40 mg	Placebo	Simvastatin 40 mg, 100%; ezetimibe 10 mg, 47%	MI, St, ReV, CVM	16%	RR 0.96 (0.90–1.03)

IMPROVE-IT: Improved Reduction of Outcomes: Vytorin Efficacy International Trial; **FOURIER:** Further Cardiovascular Outcomes Research With PCSK9 Inhibition in Subjects With Elevated Risk; **ODYSSEY:** Evaluation of Cardiovascular Outcomes After an Acute Coronary Syndrome During Treatment With Alirocumab; **CLEAR:** Cholesterol Lowering via Bempedoic acid, an ACL-Inhibiting Regimen; **ACCORD:** Action to Control Cardiovascular Risk in Diabetes; **HPS2-THRIVE:** Heart Protection Study 2-Treatment of HDL to Reduce the Incidence of Vascular Events ACS: acute coronary syndrome; ASCVD: atherosclerotic cardiovascular disease; T2DM: type 2 diabetes mellitus; MI: myocardial infarction; St: stroke; TIA: transient ischemic attack; UA-H: unstable angina requiring hospitalization; ReV: arterial revascularization procedure; CA-R: resuscitated cardiac arrest; CVM: cardiovascular mortality; ACM: all-cause mortality; UCM: unknown cause mortality.

Another PCSK9 inhibitor, bococizumab, was studied in the six parallel SPIRE investigations of the mid-2010s (total *n* = 4300). While capable of producing robust LDL-C reductions in the early period, this effect was subject to wide variability and significant attenuation by 52 weeks [56]. Almost half the patients developed anti-drug antibodies, and LDL-C reduction was attenuated in those with the highest titers [56]. In two randomized trials comparing it with placebo, bococizumab demonstrated a significant benefit with respect to major adverse cardiovascular events in higher-risk patients (HR 0.79; 95% CI, 0.65 to 0.97) [57]. In another randomized control trial, the subgroup of patients with statin-treated FH had a similar magnitude of risk reduction for major adverse cardiovascular events with bococizumab as did those with FH (HR 0.83; 95% CI, 0.55–1.54), with no evidence of statistical heterogeneity between the subgroups [58]. However, due to the high incidence of anti-drug antibody production, bococizumab was discontinued [56].

To date, the major trials of PCSK9i therapy have focused on secondary prevention, except in the case of FH. While injectable PCSK9i monoclonal antibody therapy is widely available, there is active investigation into oral PCSK9i therapy and its effects on LDL-C and major adverse cardiovascular events [59].

### 5.4 Bempedoic acid

Bempedoic acid is a prodrug, which is an ATP citrate lyase inhibitor that inhibits cholesterol synthesis in the same pathway but upstream of statins. The CLEAR Outcomes trial, a large RCT evaluating its impact on cardiovascular outcomes, randomized 13,970 high-risk primary prevention or secondary patients with self-reported statin intolerance to bempedoic acid or placebo, with a high proportion of female participants [50]. At a median follow-up of 40 months, the bempedoic acid arm demonstrated a 1.6% ARR (HR 0.87; 95% CI, 0.79 to 0.96) in the primary composite outcome. Benefits were also seen for myocardial infarction (HR 0.77) and need for coronary revascularization (HR 0.81).

A subgroup analysis of CLEAR Outcomes was published focusing on the primary prevention group, and in this analysis, a striking 39% and 27% RRR was seen for cardiovascular and all-cause mortality, respectively [60]. While these associations did meet significance, this subgroup analysis should be interpreted with some level of caution and considered hypothesis-generating [61]. CLEAR Outcomes was not powered to detect differences in subgroups, and these findings may represent chance. Notably, this was the first RCT of statin intolerant patients and demonstrates that more studies are needed in this population. Although the discussion of statin intolerance (also known as statin associated side effects) is beyond the scope of this article, a review by Bosco et al. provides an approach for the management of statin intolerant patients focusing on non-statin therapies [62].

### 5.5 Small interfering RNA against PCSK9

Inclisiran is a small interfering RNA which blocks the translation of PCSK9, and thereby acts on the same pathway as alirocumab and evolocumab, but works upstream during the translation phase rather than on the protein. It is a twice-yearly injection that has been the subject of several large RCTs in recent years. Most recently, the ORION-10 and ORION-11 studies investigated the efficacy of inclisiran versus placebo on LDL-C reduction [63]. ORION-10 randomized 1,561 patients with a history of ASCVD, while ORION-11 randomized 1,617 patients with either ASCVD or established risk equivalents [63]. The magnitude of LDL-C reduction was 52.3% and 53.8% in ORION-10 and 11, respectively [63]. Inclisiran has also been trialed in HeFH in ORION-9 with similarly impressive LDL-C reductions [64].

Notably, cardiovascular outcome reduction with inclisiran has not yet been established, but the ORION-4 trial aims to study its efficacy in the prevention of MACE in patients with known ASCVD. As of November 2023, this trial is in its recruitment phase.

## 6 Safety of very low LDL-C levels

Multiple lines of evidence, including genetic epidemiology and modern clinical trial of combination lipid lowering therapy, support the safety of very low LDL-C. In particular, the evidence from PCSK9 inhibitor trials substantially advanced our understanding of what is considered achievable and safe for LDL-C. The use of PCSK9 inhibitors in conjunction with high-intensity statins has resulted in a previously unprecedented degree of LDL-C reduction with a median LDL-C of 30 mg/dL in the FOURIER trial and 40 mg/dL by 4 months in the ODYSSEY-OUTCOMES trial [52,54]. Despite these very low LDL-Cs in the clinical trials, results have been reassuring from a safety standpoint with respect to neurocognitive disorders, hemorrhagic stroke, worsening glycemia, and/or new onset-diabetes mellitus [65], [66], [67].

Several prespecified safety analyses were performed alongside FOURIER. One such analysis evaluated 25,982 out of the 27,564 patients initially randomized to either evolocumab or placebo and found that 21% of the evolocumab arm achieved an LDL-C of less than 19 mg/dL at 4 weeks [67]. A further 59% and 11% achieved an LDL-C of 19–50 mg/dL and 50–70 mg/dL respectively, and these responses were sustained over a follow-up of 3.2 years [67]. In this timeframe, across the 10 safety endpoints studied, lower versus higher LDL-C was not associated with any differences in safety endpoints [67]. Conversely, the primary clinical outcome as well as the secondary outcome both displayed highly significant linear reductions with further LDL-C reduction [67]. These findings were redemonstrated in a safety analysis of the FOURIER-OLE study with >8 years of follow up [65]. A second analysis focused specifically on diabetes and glycemia found that the ARR of the primary outcome was, predictably, greater in diabetic patients due to their increased ASCVD risk [66]. It also found no increased incidence of diabetes or worsening glycemia in the evolocumab arm [66].

Therefore, PCSK9i trial data have provided strong reassurance with respect to the safety of very low LDL-C. Furthermore, they have led to revisiting of prior concerns from older studies. For example, although prior epidemiological studies have found an association between low serum cholesterol level and increased risk of hemorrhagic stroke, this has been an area of uncertainty. Most data from randomized clinical trials and genetic studies do not support such a link [68], [69], [70], [71], [72]. Safety analyses of statin and PCSK9i trials including FOURIER and ODYSSEY, as well as genetic conditions resulting in very low LDL-C, have provided reassurance that lowering LDL-C to low levels does not increase risk of hemorrhagic stroke [73].

## 7 Additional LDL-C lowering therapies for homozygous familial hypercholesterolemia (HoFH)

### 7.1 Lomitapide

Lomitapide is a small molecule benzimidazole that was developed as an orphan drug for the treatment of HoFH [74]. In addition to a low-fat diet and other lipid-lowering therapies, it is indicated as an adjunctive treatment for the management of HoFH [75]. It achieves its effects by inhibiting microsomal triglyceride transfer protein (MTP), which is required for the hepatic production of VLDL and chylomicrons in enterocytes [74]. In doing so, it significantly reduces the serum levels of all lipoprotein fractions, including VLDL and its downstream product, LDL [74].

In a single-arm, open-label, phase 3 study of lomitapide for treatment of patients with HoFH (*n* = 29), LDL-C was reduced by 50% (95% CI −62 to −39) from baseline [76]. Concentrations of LDL-C remained reduced by 44% (95% CI −57 to −31; *p* < 0.0001) at week 56 and 38% (−52 to −24; *p* < 0.0001) at week 78 [76]. The median dose used was 40 mg, however, it has been shown to achieve equivalent LDL-C lowering effects or greater at lower doses [77]. Although lomitapide is linked to an increase in hepatic steatosis, a study aiming to explore the long-term hepatic safety of lomitapide found no clinically significant elevations in hepatic biomarkers, and hepatic stiffness remained normal for over 9-years of follow-up [78].

### 7.2 Evinacumab

**Fig. 2:**
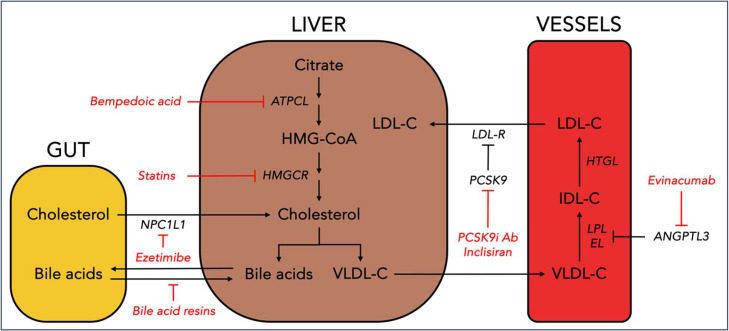
**Targets of LDL-C lowering therapies.** The figure highlights targets of LDL-C lowering therapies in the gut, liver, and vasculature.

NPC1L: Niemann-Pick C1-like 1; ATPCL: ATP citrate lyase; HMGCR: HMG-CoA reductase; LDL-R: low-density lipoprotein receptor; PCSK9: proprotein convertase subtilisin/kexin type 9; PCSK9i Ab: proprotein convertase subtilisin/kexin type 9 inhibitor antibody; ANGPTL3: angiopoietin-like 3; LPL: lipoprotein lipase; IDL-C: intermediate-density lipoprotein-cholesterol; LDL-C: low-density lipoprotein cholesterol.

Evinacumab is a monoclonal antibody against ANGPTL3, a novel target in lipid management. Unlike statins, ezetimibe, PCSK9 inhibitors, and bempedoic acid, evinacumab lowers LDL-C via a mechanism independent of the LDL receptor. ANGPTL3 is a hormone which inhibits lipoprotein and endothelial lipase (Fig. 2). Its inhibition by evinacumab appears to upregulate VLDL remnant production and clearance, and thereby reduces LDL-C as there is less substrate for LDL. It has demonstrated LDL-C reductions near those achievable with high-intensity statin therapy and PCSK9i therapy in both primary and secondary prevention patients [79]. Those who are not at goal despite optimal statin and PCSK9i therapy, or those whose hyperlipidemia is driven by an LDL-C receptor independent mechanism may be considered for evinacumab.

To date, few phase 3 clinical trials for evinacumab have been performed. ELIPSE-HoFH randomized to 65 patients with HoFH to either evinacumab 15 mg/kg or placebo [80]. Injections were administered every 4 weeks, and patients were followed for 24 weeks with a primary outcome of LDL-C reduction. Evinacumab led to a 49% reduction versus placebo. In another trial in patients with refractory hypercholesterolemia, it significantly reduced LDL-C levels by over 50% at the maximum dose [79]. Evinacumab is currently FDA approved for HoFH. Additional, outcomes-based clinical trials would be necessary to broaden its use in ASCVD prevention.

## 8 Supplements and older drugs that have fallen out of favor

Over 50% of the US population reports using supplements, and many believe that they are equally or more effective than FDA approved pharmacologic agents [81]. Some of these dietary supplements include omega-3 fatty acids and red yeast rice. Although this review does not aim to discuss these supplements, clinicians should be aware of their popularity amongst their patients. Grant et al. provides an evidence-based narrative review of dietary supplements in lipid lowering, a framework for managing expcetations, and generally encourages the use of the evidence-based therapies discussed above instead of supplements [81].

Several therapies which had previously been extensively utilized for LDL-C lowering in the pre-statin era have since fallen out of favor, including niacin and bile acid sequestrants. Niacin is a water-soluble vitamin which primarily increases HDL-C while modestly reducing LDL-C and TG, which similarly showed benefit as a monotherapy but not as an adjunct to statins [82,83]. Bile acid sequestrants reduced LDL-C by shunting the hepatic cholesterol pool away from lipoprotein synthesis. Pre-statin trials demonstrated an ASCVD benefit when utilized as a monotherapy, and subsequent trials noted incremental reduction in LDL-C with the addition of colesevelam to background statin therapy [84,85]. However, with the advent of more powerful statin adjuncts such as ezetimibe and PCSK9 inhibitors, their utilization has become less frequent.

## 9 Emerging therapeutic approaches and targets

### 9.1 Gene-Editing technologies targeting PCSK9

Research is underway on several cellular and gene therapies which can modify or silence PCSK9 at the genetic level. Models in mice (1) and primates (2, 3, 4) have demonstrated successful editing of the PCSK9 gene through epigenetic modulation, meganucleases, base editors, and the CRISPR-Cas9 system [86], [87], [88], [89], [90]. Significant reductions in PCSK9 expression and LDL-C levels have been noted, with similar findings recently found in humans as well [91]. If continued development of gene therapies is able to establish safety and efficacy, it could be a transformative approach in clinical practice due to the potential for a highly durable effect on LDL-C lowering.

### 9.2 Lp(a)

Elevated lipoprotein a (Lp(a)) is a genetic risk factor that is causally linked to ASCVD. It is composed of an LDL-like particle in which apoB is covalently bound by a single disulfide bond to apolipoprotein (a), which is the pathognomonic component of Lp(a) [92]. As such, it is being targeted for drug development, with several agents in the pipeline [93]. AKCEA-APO(a)-LRx, now known as pelacarsen, is an antisense oligonucleotide that targets and reduces the production of apo(a). It targets LPA mRNA and is conjugated with triantennary N-acetylgalactosamine, which directs therapy specifically to hepatocytes. This novel drug induces ∼80% reductions in Lp(a) levels compared with placebo [94]. Another modality of targeting Lp(a) includes siRNA. Olpasiran is an *N*-acetylgalactosamine (GalNAc)-conjugated siRNA that was modified with 2′-fluoro and 2′‑methoxy substitutions and phosphorothioate internucleotide linkages at the termini [95]. In a randomized control trial with 281 patients with established cardiovascular disease, it was found to significantly reduce Lp(a) concentrations in a dose-dependant manner [96]. In a randomized control trial aiming to study an oral small molecule inhibitor of Lp(a) called muvalaplin, 114 participants were randomized to either a single-ascending dose, or a multiple-ascending dose group [97]. It was found to lower plasma Lp(a) levels by 63% - 65%, resulting in levels less than 50 mg/dL in 93% of participants, and was not associated with tolerability concerns [97]. A phase 1, single-ascending dose trial studying lepodisiran, an extended-duration short interfering RNA targeting Lp(a), enrolled 48 adults elevated Lp(a) and no cardiovascular disease [98]. Lepodisiran was found to be well tolerated and produced dose-dependent, long duration reductions in Lp(a) concentrations, with a median change of 94% at day 337 in the 606 mg dose group [98]. Multiple cardiovascular outcome trials are now underway.

### 9.3 CETP

Cholesterol ester transfer protein (CETP) inhibitors are a class of drugs whose lipid-lowering was initially focused on HDL-C raising properties, but recent development has shifted to LDL-C lowering. In early efforts, CETP inhibitors had adverse off-target and unpredictable events such as hypertension, reduction in glomerular filtration rate, and electrolyte changes [99]. The ILLUMINATE study which tested Torcetrapib, was stopped prematurely due to an increased risk of death in treated patients. Earlier trial data from ACCELERATE which tested Evacetrapib was also negative. Recently however, the REVEAL trial which tested Anacetrapib, has indicated a benefit related to LDL-C lowering [99]. The oral CETP inhibitor Obicetrapib has stronger LDL-C lowering than prior CETP inhibitors and a promising safety profile. In the ROSE2 trial, when added to high-intensity statin therapy, LDL-C reductions of 63.4%, 43.5%, and 6.35% were seen in the combination Obicetrapib and ezetimibe group, Obicetrapib monotherapy group, and placebo group, respectively [100]. Obicetrapib is currently undergoing evaluation in clinical outcomes trials.

## 10 Cost, affordability, and potential challenges with access

Whereas generic options exist for statins and ezetimibe, the cost of other non-statins poses a challenge in affordability and equitable access to optimal LDL-C lowering therapy. Analysis of claims for PCSK9i therapy has shown that in the first year of their availability, only 47% of prescriptions were ever approved [101]. Even for patients who appear to meet the labeled indications for PCSK9i, rates of approval are low [102]. The friction from low approval rates and the administrative burden of the prior authorization process makes patient access to therapy more challenging, and even when approval is obtained, high co-payments can limit access to therapy. While patient assistance programs may help, there is a need for continued innovation in drug coverage to facilitate the broad and equitable adoption of modern lipid lowering therapies in patients who can benefit.

## 11 Conclusion

Cardiovascular risk management continues to evolve as novel mechanisms of modifying risk in atherosclerosis emerge. Early, intensive, and sustained LDL-C lowering is central in the primary and secondary prevention of ASCVD. The discovery of emerging novel targets has led to the development of several drug therapies that present new, promising frontiers for LDL-C reduction, especially in populations whose elevated cardiovascular risk has proven challenging to optimize. We have learned that lower for longer is better, and achieving very low LDL-C is feasible and safe. As further advances are made in novel drug development, efforts must continue to focus on translating research into practical, accessible, and cost-effective strategies with the goal of improving patient-centered care and outcomes.

## Disclosures

OM, ZAB, and SLS have no disclosures to report. PK: Speaker's Bureau: Boston Scientific, Amarin, Esperion, AstraZeneca, Merck, Advisory Board: Amgen, Boston Scientific, Novartis, Esperion, Doximity, DocWire/MashUP MD, Honoraria: ACC/ABIM Question Writing, Current Atherosclerosis Reports Section Editor, Amgen, Consultant: Grand Rounds, American College of Cardiology, Grand Rounds, 2nd MD, Writing/Editorial Board: Healthline, GE/SkyWord, American College of Cardiology. Under a license agreement between Corrie Health and Johns Hopkins University, the university owns equity in Corrie Health. The university, FAM and SSM are entitled to royalty distributions related to Corrie Health. In addition, FAM and SSM are co-founders of and hold equity in Corrie Health. This arrangement has been reviewed and approved by Johns Hopkins University in accordance with its conflict of interest policies. FAM and SSM have also received research and material support from Apple and iHealth. Furthermore, SSM is on the Advisory Board for Care Access and reports personal consulting fees from Amgen, AstraZeneca, BMS, Chroma, Kaneka, NewAmsterdam, Novartis, Novo Nordisk, Premier, Sanofi, and 89bio. Outside this work, SSM reports research support from the American Heart Association (20SFRN35380046, 20SFRN35490003, #878,924, #882,415, #946,222), the Patient-Centered Outcomes Research Institute (ME-2019C1–15 328, IHS-2021C3–24,147), the National Institutes of Health (NIH) (P01 HL108800 and R01AG071032), the David and June Trone Family Foundation, the Pollin Digital Innovation Fund, Sandra and Larry Small, Google, and Merck.

## Declaration of competing interest

The authors declare the following financial interests/personal relationships which may be considered as potential competing interests: OM, ZAB, and SLS have no disclosures to report. PK: Speaker's Bureau: Boston Scientific, Amarin, Esperion, AstraZeneca, Merck, Advisory Board: Amgen, Boston Scientific, Novartis, Esperion, Doximity, DocWire/MashUP MD, Honoraria: ACC/ABIM Question Writing, Current Atherosclerosis Reports Section Editor, Amgen, Consultant: Grand Rounds, American College of Cardiology, Grand Rounds, 2nd MD, Writing/Editorial Board: Healthline, GE/SkyWord, American College of Cardiology. Under a license agreement between Corrie Health and Johns Hopkins University, the university owns equity in Corrie Health. The university, FAM and SSM are entitled to royalty distributions related to Corrie Health. In addition, FAM and SSM are co-founders of and hold equity in Corrie Health. This arrangement has been reviewed and approved by Johns Hopkins University in accordance with its conflict of interest policies. FAM and SSM have also received research and material support from Apple and iHealth. Furthermore, SSM is on the Advisory Board for Care Access and reports personal consulting fees from Amgen, AstraZeneca, BMS, Chroma, Kaneka, NewAmsterdam, Novartis, Novo Nordisk, Premier, Sanofi, and 89bio. Outside this work, SSM reports research support from the American Heart Association (20SFRN35380046, 20SFRN35490003, #878,924, #882,415, #946,222), the Patient-Centered Outcomes Research Institute (ME-2019C1–15 328, IHS-2021C3–24,147), the National Institutes of Health (NIH) (P01 HL108800 and R01AG071032), the David and June Trone Family Foundation, the Pollen Digital Innovation Fund, Sandra and Larry Small, Google, and Merck.
